# Developments and Applications of Molecularly Imprinted Polymer-Based In-Tube Solid Phase Microextraction Technique for Efficient Sample Preparation

**DOI:** 10.3390/molecules29184472

**Published:** 2024-09-20

**Authors:** Hiroyuki Kataoka, Atsushi Ishizaki, Keita Saito, Kentaro Ehara

**Affiliations:** School of Pharmacy, Shujitsu University, Nishigawara, Okayama 703-8516, Japan

**Keywords:** molecularly imprinted polymer (MIP), in-tube solid-phase microextraction (IT-SPME), sample preparation

## Abstract

Despite advancements in the sensitivity and performance of analytical instruments, sample preparation remains a bottleneck in the analytical process. Currently, solid-phase extraction is more widely used than traditional organic solvent extraction due to its ease of use and lower solvent requirements. Moreover, various microextraction techniques such as micro solid-phase extraction, dispersive micro solid-phase extraction, solid-phase microextraction, stir bar sorptive extraction, liquid-phase microextraction, and magnetic bead extraction have been developed to minimize sample size, reduce solvent usage, and enable automation. Among these, in-tube solid-phase microextraction (IT-SPME) using capillaries as extraction devices has gained attention as an advanced “green extraction technique” that combines miniaturization, on-line automation, and reduced solvent consumption. Capillary tubes in IT-SPME are categorized into configurations: inner-wall-coated, particle-packed, fiber-packed, and rod monolith, operating either in a draw/eject system or a flow-through system. Additionally, the developments of novel adsorbents such as monoliths, ionic liquids, restricted-access materials, molecularly imprinted polymers (MIPs), graphene, carbon nanotubes, inorganic nanoparticles, and organometallic frameworks have improved extraction efficiency and selectivity. MIPs, in particular, are stable, custom-made polymers with molecular recognition capabilities formed during synthesis, making them exceptional “smart adsorbents” for selective sample preparation. The MIP fabrication process involves three main stages: pre-arrangement for recognition capability, polymerization, and template removal. After forming the template-monomer complex, polymerization creates a polymer network where the template molecules are anchored, and the final step involves removing the template to produce an MIP with cavities complementary to the template molecules. This review is the first paper to focus on advanced MIP-based IT-SPME, which integrates the selectivity of MIPs into efficient IT-SPME, and summarizes its recent developments and applications.

## 1. Introduction

Although state-of-the-art high-performance analytical instruments with improved sensitivity and selectivity have been developed for qualitative and quantitative analysis of analytes in complex matrices, sample preparation remains a time-consuming task for researchers [1,2,3,4,5,6,7,8,9,10]. This step is often considered the bottleneck of the entire analytical process, and it is no exaggeration to say that the efficiency of sample preparation greatly affects the quality of the analytical results. For instance, in most chromatographic systems, target analytes present at trace levels in complex matrices may coelute with other structurally similar compounds that coexist at high concentrations [5]. Additionally, coexisting biological macromolecules may adsorb irreversibly to the inner walls of tubing or the pores of the column’s stationary phase, causing flow path blockages and decreased column efficiency [3]. In mass spectrometry, ionization suppression by the matrix can significantly impact detection sensitivity and reproducibility. Consequently, sample pretreatment is an essential and critical step not only for pre-separating and enriching the target analyte, but also for removing interfering components, enhancing detection, eliminating matrix interference, improving analytical sensitivity and accuracy, and reducing instrument maintenance and operating costs.

Various sample preparation methods have been used [1,2], but classical liquid-liquid extraction (LLE) [11] requires large volumes of samples and toxic organic solvents, and is time-consuming, labor-intensive, and costly. Solid-phase extraction (SPE) [12,13,14] was developed to overcome these drawbacks and is widely used due to its relatively simple and efficient operation, low cost, reduced consumption of organic solvents, and high enrichment capability. Furthermore, to minimize sample size and solvent use, and to improve efficiency and automation, various microextraction techniques have been developed, including micro-SPE [15], dispersive micro-SPE [16], solid-phase microextraction (SPME) [17,18,19,20,21], stir bar sorptive extraction (SBSE) [22,23,24], micro extraction in a packed syringe [25], pipette tip SPE [26], magnetic bead extraction [27] and liquid-phase microextraction [28,29,30]. Among these, in-tube SPME (IT-SPME) [3,4,5,31,32,33,34,35,36,37], which uses a capillary column as the extraction device, is particularly useful. IT-SPME requires almost no organic solvent, resulting in less liquid waste, higher throughput, smaller size, online connection to analytical instruments, and automation that saves labor and allows continuous overnight operation. As such, IT-SPME is an advanced sample preparation method that aligns well with the principles of green analytical chemistry.

However, the performance of these early microextraction methods is often dependent on the matrix composition, as they are limited in increasing extraction volumes due to sample loading constraints and may be ineffective in selectively extracting analytes from coexisting substances. To improve extraction efficiency and selectivity, various new functional adsorbents have been developed, including monoliths [38], ionic liquids (ILs)/polymer ILs [39], restricted-access materials (RAMs) [40], molecularly imprinted polymers (MIPs) [6,7,8,9,10,41,42,43,44,45,46,47,48,49], graphene/graphene oxide [50,51,52], carbon nanotubes (CNTs) [53], inorganic nanoparticles (NPs) [54,55], metal organic frameworks (MOFs) [56,57], and covalent organic frameworks (COFs) [58,59]. Among these, MIPs, chemically stable polymers that acquire template cavities during synthesis, have superior molecular recognition capabilities for specific analytes or structural analogues, making them “smart adsorbents” with high selectivity and loading capacity. Molecular imprinting technology has thus become a powerful tool in the development of advanced sample preparation methods.

Although many excellent review papers have discussed SPE [60,61,62] and SPME [8,9,10,63,64,65,66] using MIP in terms of adsorbents and extraction methods, there has been no review focused on MIP IT-SPME, which integrates MIP into the IT-SPME method. In this review of recent developments and applications of MIP IT-SPME, we focus on MIP IT-SPME, which combines the characteristics of both methods, such as simplicity, flexibility, robustness, and selectivity.

## 2. Overview of IT-SPME

IT-SPME, developed by Eisert and Pawliszyn [67] in 1997, is a microextraction technique designed for efficient sample preparation using open tubular fused silica capillary columns as extraction devices. This technique addresses several drawbacks of the initial SPME [68], which uses fused silica fibers with sorbent coatings on the surface as extraction devices. The limitations of the original approach included: (1) fiber fragility, (2) bleeding from the thick film coating, (3) low adsorption capacity, (4) difficulty in applying the technique to non-volatile and thermally unstable compounds unsuitable for gas chromatography (GC) or GC-mass spectrometry (MS), and (5) low stability in the presence of solvents used in high-performance liquid chromatography (HPLC). IT-SPME is a dynamic in-flow microextraction technique that is particularly useful for automated clean-up and rapid on-line coupling to liquid chromatography (LC) through column switching [1,2,3,4,5,31,32,69,70]. Analytes in complex matrices can be continuously analyzed by HPLC and MS/MS systems linked to IT-SPME with minimal processing such as filtration.

### 2.1. IT-SPME Operating System

**Figure 1:**
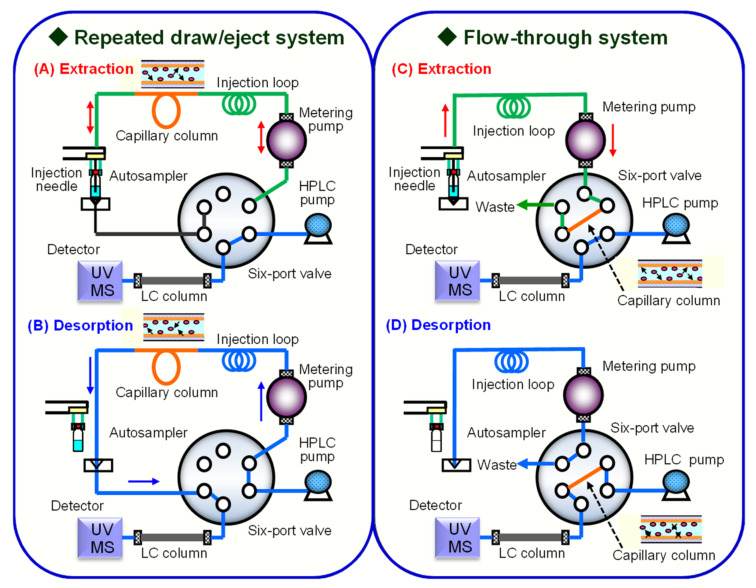
Two operating systems of automated online IT-SPME coupled with HPLC. (**A**,**C**) are the steps of extracting compounds from the sample solution into the capillary stationary phase, and (**B**,**D**) are the steps of desorbing the compounds extracted into the capillary. The green and blue lines indicate the flow of the sample solution and the mobile phase, respectively. Reproduced from Figure 5 of Ref. [3] with permission from Elsevier.

In IT-SPME, operations like extraction, concentration, desorption, and injection can be easily automated using a programmable autosampler and column switching technology [3,32,33]. First, as the sample solution passes through the extraction capillary, the analyte is extracted and concentrated by adsorption or absorption onto the stationary phase in the capillary, based on the distribution equilibrium. For samples with high levels of coexisting substances, water or an appropriate solvent can be passed through the capillary after loading the sample to selectively wash away unwanted matrix components. The extracted analyte is then desorbed either by introducing a solvent into the capillary via valve switching (static desorption) or through a mobile phase (dynamic desorption) and transferred to the analytical instrument. These operating systems include the draw/eject system, where the sample solution is repeatedly aspirated and discharged into the capillary, and the flow-through system, in which sample solution flows in one direction into the capillary (Figure 1). In the repeated draw/eject system, the amount of adsorption depends on the distribution rate to the capillary’s stationary phase and the number of repetitions, since the total amount of compounds in the solution is not fully loaded by repeated extractions from a fixed volume. In contrast, in the flow-through system, the amount of adsorption increases with the volume of the sample solution, but adsorption in a narrow capillary is limited by the thickness of the coating and the distribution equilibrium of the compounds, which may lead to overflow. Other innovative approaches to improve extraction and desorption efficiency include magnetic IT-SPME [71,72], which utilizes magnetic nanomaterial-filled tubes to perform extraction and desorption by switching magnetic fields, electrochemically controlled IT-SPME [73,74,75], and IT-SPME with temperature control devices [76,77].

IT-SPME offers the advantage of enabling high-throughput analytical systems through process automation and online coupling with analytical instruments. It is primarily used in direct connection with HPLC, but it can also be integrated [78,79,80,81] with capillary electrophoresis, capillary electrochromatography, direct MS, or atomic absorption spectrometry. Recently, the power of LC has increased, and coupling with miniaturized chromatography systems, such as ultra-high-pressure LC (UHPLC), capillary LC (capLC), or nanoLC, facilitates integration with MS detectors, enhances column efficiency and sensitivity, reduces solvent consumption, and shortens analysis time. However, online coupling with these miniaturized systems requires specialized interfaces to control flow rates.

### 2.2. Capillary Tube Configuration for IT-SPME

**Figure 2:**
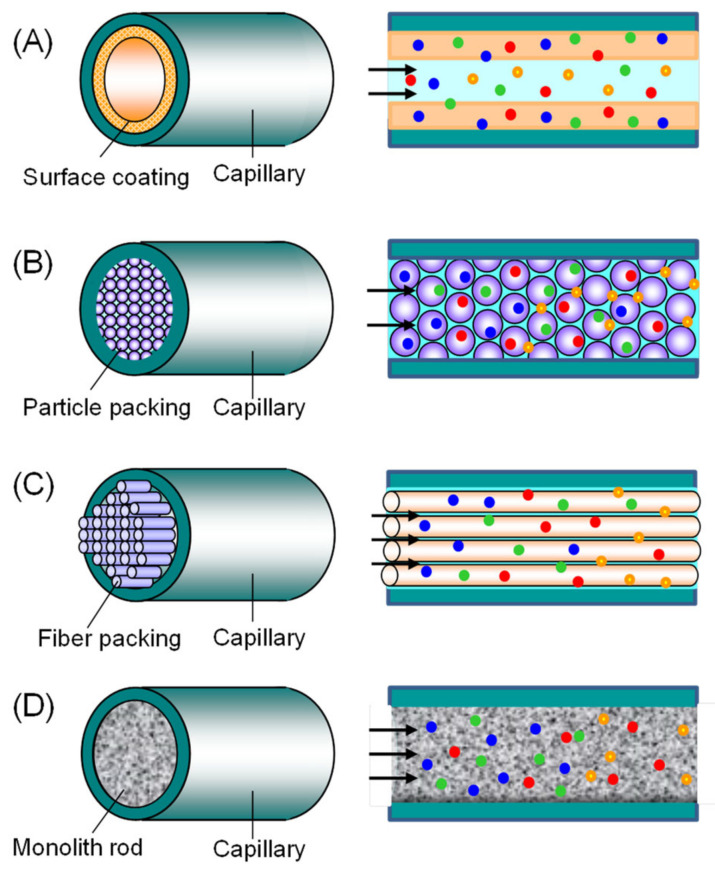
Configurations of capillary tubes for IT-SPME. (**A**) Inner-surface-coated capillary, (**B**) particle-packed capillary, (**C**) fiber-packed capillary, (**D**) monolithic capillary. Reproduced from Figure 4 of Ref. [3] with permission from Elsevier.

Capillary tube configurations used in IT-SPME can be classified into four types (Figure 2): (1) inner-surface-coated, (2) particle-packed, (3) fiber-packed, and (4) rod monolith capillaries [3,4,33]. The details are as follows:

1. Inner-surface-coated: includes wall-coated open tubular (WCOT) capillaries and porous layer open tubular (PLOT) capillaries, where the inner wall of the tube is coated with an adsorbent.
2. Particle-packed: capillaries where adsorbent-coated particles are packed inside the tube.
3. Fiber-packed: capillaries in which thin fibers are vertically packed inside the tube.
4. Rod monolith: capillaries where a monolith is formed within the tube.

Open-tube capillaries offer the advantage of avoiding clogging without increasing the tube’s back pressure, even at faster flow rates compared to packed capillaries. They are versatile, interacting with a variety of compounds [3]. However, commercially available GC capillary columns have limitations in selectively extracting and concentrating specific target analytes from complex matrices due to the limited nature and dimensions of the phases, and their infrequent interaction with polar compounds.

For IT-SPME, fused silica tubes with a fixed inner diameter of 1 mm or less and an outer polyimide coating for enhanced flexibility and durability are commonly used. Other materials such as polyetheretherketone (PEEK), polytetrafluoroethylene, polydimethylsiloxane (PDMS), stainless steel, and glass tubes are also utilized [82]. Fused silica capillaries, where silanol groups on the inner wall are ionized with buffer solution, are typically used for adsorbent immobilization. The immobilized phase is formed through polymer coating, polymerization, chemical modification, or electrodeposition. In contrast, due to the difficulty of adsorbent immobilization on the inner walls of plastic or stainless-steel tubes, these materials are often used as packed tubes filled with adsorbent particles or fibers. Fiber-filled capillaries increase the adsorption surface area and improve extraction efficiency by coating the fiber surface. Porous monolithic capillaries enhance extraction efficiency through high permeability, rapid mass transfer, high stability, and high loading capacity.

In IT-SPME, the amount and sensitivity of the extracted analytes depend on the length of the extraction capillary and the adsorption capacity of the adsorbent. Generally, a longer capillary increases the amount extracted, but it also broadens the sample bandwidth, affecting subsequent chromatographic separation. This can delay the target compound’s arrival at the detector, leading to excessive peak broadening. Therefore, extraction capillaries typically range from 60 to 80 cm in length. When connecting to chromatography systems with low mobile phase flow rates, such as capLC or nanoLC, these effects should be considered. Additionally, the dimensions of other components necessary for setting up the extraction device (e.g., connectors, injection valves) should be carefully selected to prevent band broadening [32].

### 2.3. Extraction Phase of Capillary Tube

In IT-SPME, the affinity of the analyte for the extraction phase of the capillary tube affects the extraction efficiency, so selecting an appropriate extractant based on the properties of the target compound is essential. Common sorbents include carbon-based sorbents (e.g., divinylbenzene polymer (DVB), carboxene, carbon molecular sieves, polyethylene glycol, etc.) and silica-based sorbents (e.g., polydimethylsiloxane (PDMS), diphenyl-polydimethylsiloxane, cyanopropylphenylmethylpolysiloxane, etc.). These sorbents have been used since the early days of IT-SPME development because they are commercially available capillary columns for GC [3]. These sorbents are used in both hollow coated WCOT and PLOT columns. WCOT columns, such as TRB and CP-Sil 19CB, are liquid-phase types, while PLOT columns, such as Supel Q PLOT and Carboxen 1006 PLOT, are adsorption types. Liquid-phase capillaries, in which the adsorbent is firmly bonded and cross-linked to the capillary inner wall, can be used stably without losing phase when the solvent passes through the capillary. However, their film thickness cannot be made sufficiently thick to achieve a strong extraction effect. On the other hand, adsorption-type capillaries, which are porous with a large surface area, offer high extraction efficiency, but their thick film can deteriorate and exfoliate depending on the mobile phase solvent. A significant advantage of commercially available capillaries is the stable supply of products with various polarities, film thicknesses, and porosities, making them applicable to the extraction of various compounds. Additionally, they offer excellent reproducibility and reusability due to stable extraction performance. However, as mentioned above, their lack of selectivity and relatively low extraction efficiency limit their usefulness.

To improve extraction efficiency and selectivity, various functional extraction phases have been developed [3,35,36,37]. These include carbon-based nanomaterials such as CNTs and graphene, as well as monoliths, RAM, MIP, ILs, MOF, and deep eutectic solvents (DES). These materials can be coated on the capillary inner wall, or particles or fibers coated and packed in the capillary. For example, CNTs and graphene have high surface areas and provide high adsorption efficiency for target analytes due to interactions such as hydrogen bonding, π-π stacking, electrostatic forces, van der Waals forces, and hydrophobic interactions [83]. IT-SPME using octadecyl silica (C18) monolithic rod capillaries also offers higher extraction efficiency than conventional PDMS-coated capillaries and can be coupled online with capLC systems. Additionally, biocompatible RAM capillaries, MIP capillaries, and immunoaffinity capillaries packed with alkyl diol silica (ADS) particles have been developed to improve selectivity [3,8,9,10,31,35,36,37,64,65]. MIPs, in particular, are synthetic polymeric materials that consist of complementary imprinted moieties for specific molecules. They recognize target analytes and compounds with similar molecular structures through a combination of hydrogen bonding, hydrophobicity, and electrostatic interactions, resulting in high extraction selectivity. ILs, MOFs, and DES have also been applied as extractants for IT-SPME. Hybrid materials combining extractants with different functions, such as RAM and MIP, MOF embedded in monoliths, and graphene monoliths embedded in porous polymers, are also promising adsorbents. Highly porous nanoparticle coatings with magnetic hybrid extractant phases have been applied in magnetic IT-SPME as well [70,71]. This review focuses on MIPs, known for their high molecular recognition capacity, which are summarized in detail in the following sections.

## 3. Fabrication of Molecularly Imprinted Polymers

MIPs were first developed by Wulff [84] and Mosbach [85] in the 1970s to recognize the shape, size, and functional groups of specific target molecules, such as template molecules and their structural analogues. These artificial functional materials mimic biological interactions, such as enzyme-substrate, antigen-antibody and hormone-receptor interactions [6,47]. MIPs are characterized by their unique structural predictability, high specificity and retention, physical and chemical robustness, reusability, and batch-to-batch recognition reproducibility. Their many advantages include relatively simple preparation, low cost, and resistance to high temperature, pressure, acids, bases, organic solvents, and biological degradation [21,62,64,65]. Due to these properties, MIPs are prepared in coated particles, coated fibers, coated stir bars, coated thin films, wall-coated capillaries, monoliths, magnetic beads, and used in various device forms, including SPE [60,61,62], SPME [8,9,10,63,64,65,66] (fiber, in-tube, monolith, dispersion particles, membranes), SBSE, and others, offering superior functionality.

### 3.1. Principles of Molecular Imprinting and Synthesis of MIPs

**Figure 3:**
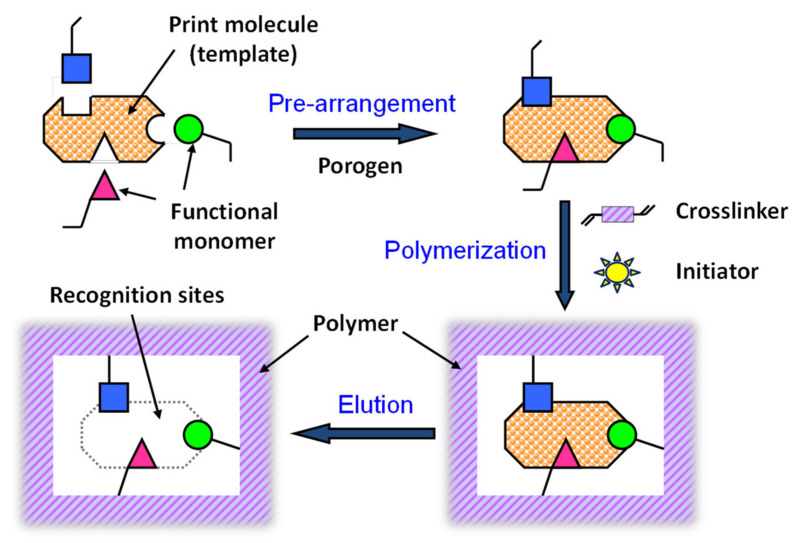
Fabrication process of molecularly imprinted polymer.

The fabrication process of MIPs generally involves three main steps: prearrangement, polymerization, and template elution (Figure 3) [6,7,8,9,10,21,41,42,43,44,47,60,64,65,66].

1. Prearrangement: In the initial step, template molecules interact with functional monomers through non-covalent, covalent, or semi-covalent bonds, forming a host-guest complex. In non-covalent imprinting, the template and monomer form a monomer-template complex via non-covalent bonds (electrostatic interactions, hydrogen bonds, ion pair formation, van der Waals forces, π-π stacking, metal coordination, etc.). This method facilitates the removal of the template without chemical bond cleavage, preserving the polymer structure. However, the stability of these bonds can be sensitive to changes in the chemical environment, requiring careful optimization of reaction conditions. Excessive use of functional monomers may also introduce non-specific binding sites, reducing selectivity. In contrast, covalent imprinting involves reversible covalent bonding between the template and polymerizable groups. After polymerization, the template is cleaved, leaving functional groups correctly orientated for subsequent re-binding. Although this method requires suitable template-monomer complexes, it ensures accurate uptake of target analytes from aqueous solutions. However, the formation and cleavage of covalent bonds are unlikely to occur under mild conditions. Semi-covalent imprinting combines covalent and non-covalent interactions, offering a balance between template stability and re-binding efficiency.
2. Polymerization: Polymerization is initiated by thermal or UV activation in the presence of cross-linkers and initiators, forming a highly cross-linked polymer network around the template molecules. This step creates the three-dimensional space necessary for molecular recognition.
3. Template elution: In the final step, template molecules are removed from the polymer network through physicochemical methods such as hydrolysis or desorption, leaving MIPs with cavity sites complementary to the template molecules. However, in covalent imprinting, removal of the template by covalent bond cleavage under severe conditions may affect the functionality of the cavity.

Among these methods, non-covalent imprinting is the most popular due to the availability of a wide variety of monomers that interact with different templates [64]. Although it requires time and effort for optimization, non-covalent imprinting allows easy template removal and re-binding under mild conditions. To synthesize non-covalent MIPs with appropriate recognition properties, mechanical and chemical stability, various factors must be optimized, including the chemistry and relative amounts of polymer components (templates, monomers, cross-linkers, initiators, porogens) and polymerization conditions (temperature, initiator activation, etc.) [9,10,18,43,47,62,64]. In trace analysis, when residual templates can cause positive errors, a dummy template—a structurally similar molecule—is used. The ideal dummy template should replicate the shape and functional group orientation of the analyte-template complex. The choice of functional monomers is closely related to the nature of the template, and the monomers must be properly selected to obtain optimal MIP functionality for the target analyte. Acidic monomers are suitable for basic templates and basic monomers for acidic templates, and methacrylic acid (MAA) and 4-vinylpyridine (VP) are commonly used as acidic and basic monomers, respectively. Cross-linkers, such as ethylene glycol dimethacrylate (EGDMA) and trimethylolpropane trimethacrylate (TRIM) play an important role in stabilizing the three-dimensional network of molecular recognition sites and controlling polymer porosity. They affect surface polarity (wettability), area, pore size, and adsorption capacity, but the relationship between adsorption capacity and crosslinker loading is complex affecting the accessibility of the binding site. To obtain a rigid polymer, the minimum required cross-linker must be used, but too much can make the structure too rigid or fill the pore structure, resulting in poor rates of template removal and re-binding. The initiator, typically azobisisobutyronitrile (AIBN), is selected based on the type of polymerization reaction, and the porogen used to solubilize the polymer components influences the physical properties of the MIP, including recognition, enantioselectivity, surface area, pore volume, and swelling. When the main template-monomer interaction is hydrogen bonding, hydrogen bond donors and non-polar, non-protic solvents with low capacity as acceptors are suitable for non-covalent imprinting.

MIPs are synthesized using various imprinting techniques, including surface imprinting, nanoimprinting, living/controlled radical polymerization, multi-template imprinting, multifunctional monomer imprinting, and dummy template imprinting (Table 1) [6]. Common polymerization methods include bulk polymerization, suspension polymerization, emulsion polymerization, precipitation polymerization, multistep swelling polymerization, sol-gel polymerization, multistep swelling polymerization, and in-situ polymerization [6,9,21,41,43,44,45,46,47,62,64,86]. Bulk polymerization, while common, may destroy binding sites during grinding and produce irregularly shaped particles. Precipitation polymerization, based on the growth of polymer chains, and suspension polymerization, which occurs in micelles, yield particles with more regular shapes [43,47]. On the other hand, sol-gel polymerization can be carried out in aqueous media under mild thermal conditions. The resulting MIP has a high degree of cross-linking and excellent thermal and mechanical stability. Therefore, it is useful for forming thin films as an adsorbent with controlled pore size and surface area [41,46].

### 3.2. Characteristics and Functionalization of MIPs

One of the main advantages of MIPs is that their customizable, allowing specific functional groups to be incorporated and external surface properties to be easily tuned [21,62]. Hybrid MIPs, which combine other extractive phases like RAM and MOF with MIP, have been developed to enhance performance [18]. For example, RAM-MIP adsorbents, made with hydrophilic monomers such as 2-hydroxyethyl methacrylate and glycerol dimethacrylate, selectively exclude macromolecules due to the hydrophilic action of the restricted-access outer layer of RAM, allowing small molecular target analytes to penetrate and be selectively extracted within the MIP phase. This reduces the need for pretreatment, such as protein precipitation, in analytic protocols. MIP-MOF hybrids, where MOF provides a robust polymer structure, increase the surface area and porosity of the MIP, facilitating mass transfer of the target analyte to the binding site [65,66]. MIP adsorbents can also be synthesized with reduced particle size without compromising specificity and selectivity, enhancing reusability and reducing analytical costs. Divinylbenzene cross-linked MIPs exhibit long-term stability and can be reused over 100 times without losing functionality, even under harsh conditions such as high acidity and temperature. However, MIPs derived from acrylates or methacrylates may exhibit a reduced adsorption specificity and capacity due to irreversible hydrolysis or esterification of the polymer regions, particularly under harsh conditions, limiting reusability.

Challenges affecting MIP-based microextraction include template leakage, binding site heterogeneity, delayed analyte diffusion to imprinted sites due to broad pore size distribution, and reduced selective binding capacity in aqueous media [65,66]. Various MIPs have been developed to address these issues. Dummy templates help avoid template leakage, while alternative polymerization methods produce uniform MIP particles, such as spherical particles and monolithic imprinted materials, with improved mass transfer rates [62]. In situ polymerization of MIP monoliths offers lower back pressure and controlled porosity, while precipitation and emulsion polymerization yield microspheres or nanospheres with large specific surface areas, speeding up template removal and improving adsorption and desorption rates. Core-shell materials, such as those combining siloxane cores with MIP shells, have enabled precise control of adsorbate morphology, enhancing the adsorption process. Additionally, CNTs, MWNTs, and magnetic nanoparticles have been used as supports in surface imprinting methods. Core-shell magnetic MIPs (MMIPs) are easy to prepare, chemically stable, and have high binding rates due to their small size and high surface area-to-volume ratio [65]. These materials offer the ability to bind to other components and integrate magnetic properties, not only by use of magnetite, but also cobalt-based magnetic nanoporous carbon, making them useful for selective extraction from complex samples. MIP-based extraction methods have also focused on increasing throughput and efficiency, leading to the development of single, dual, and multi-template MIPs for bioanalytical applications [66].

## 4. Developments and Applications of MIP IT-SPME

As sample preparation trends towards miniaturization, ease of operation, and automation, making IT-SPME has gained attention for its compatibility with chromatographic instruments. As described in Section 2, there are four main configurations of capillary tubes used in IT-SPME (Figure 2): inner surface-coated capillaries, particle-packed capillaries, fiber-packed capillaries, and rod monolith capillaries [3,4]. These capillaries, containing various adsorbents, have been applied to analyze a wide range of compounds. Among these, the integration of MIPs with IT-SPME has particularly attracted interest, combining the selectivity of MIPs with the practicality of IT-SPME. The first MIP-based IT-SPME device was reported in 2001 [87], where a propranolol-imprinted MIP sorbent was packed into PEEK tubes. Since then, MIP-based IT-SPME methods have expanded, including techniques such as coating the inner wall of capillaries with MIP, packing capillaries with MIP-coated particles or fibers, and using MIP-formed rod-shaped monoliths. These methods have been predominantly applied to the analysis of biological samples, as summarized in Table 2.

### 4.1. Selectivity and Extraction Efficiency of MIP IT-SPME

**Figure 4:**
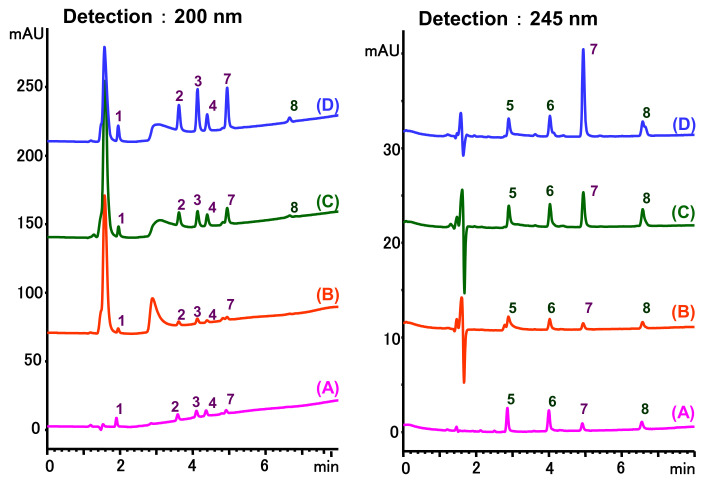
Chromatograms of steroid hormones obtained by HPLC-UV. (A) Direct injection, (B) IT-SPME using host capillary, (C) IT-SPME using NMIP, (D) IT-SPME using MIP. HPLC conditions: column, Eclipse SDB-C8 (150 × 4.6 mm ID, 5 μm particle size, Agilent Technologies, Santa Clara, CA, USA); gradient elution, acetonitrile/H_2_O (45/55) 1 mL min^−1^ → acetonitrile/H_2_O (65/35) 1.8 mL min^−1^ (8 min); column temperature, 40 °C; detection, UV at 200 and 245 nm. Peaks: 1 = estriol, 2 = β-estradiol, 3 = ethynylestradiol, 4 = diethylesilbestrol, 5 = corticosterone, 6 = testosterone, 7 = estrone, 8 = progesterone.

MIPs have been employed in IT-SPME systems to selectively recognize and extract compounds based on their template molecules. For instance, MIP capillaries were fabricated using the female hormone β-estradiol as a template, allowing for the selective extraction of estrogens. The MIP was synthesized by dissolving the template, functional monomer VP, and cross-linker EGDMA in a 1:6:30 ratio in dichloromethane and adding the polymerization initiator AIBN. The coating on the inner wall of a fused silica capillary was created by inserting fluorocarbon yarn into the capillary, filling it with the polymer preparation solution, polymerizing it at 50 °C, and then pulling the yarn out. Steroid hormones, environmental estrogens and related chemicals were analyzed using on-line IT-SPME HPLC-UV. The extraction effects were compared using the direct injection method, and IT-SPME methods with uncoated host capillaries, non-imprinted (NMIP) capillaries prepared without templates, and MIP capillaries. As shown in Figure 4, among the steroid hormones, natural and synthetic female hormones were selectively extracted and enriched. For various estrogens and related environmental chemicals, the imprint factor (IF: peak area ratio obtained using MIP and NMIP capillaries, MIP/NMIP) and the enrichment factor (EF: peak area ratio obtained using MIP IT-SPME and direct injection methods, MIP/Direct) were compared. Table 3 shows that molecular recognition ability was high for female hormones but lower for other steroid hormones. Compounds like genistein and bisphenol showed similar IFs, but IFs for nonylphenol and phthalate esters were low. These results suggest that compounds with phenolic hydroxyl groups and polycyclic skeletons are selectively recognized by MIP. Enrichment factors were also high for molecularly recognized compounds, but PCBs and DDT also had high EFs as well, indicating non-selective adsorption due to hydrophobic interactions with the polymer. Therefore, while MIP can extract and concentrate compounds similar in structure to the template in a group-selective manner, non-selective adsorption on the polymer itself must be considered.

### 4.2. Fabrications of Various MIP Capillaries and Their Applications to IT-SPME

**Figure 5:**
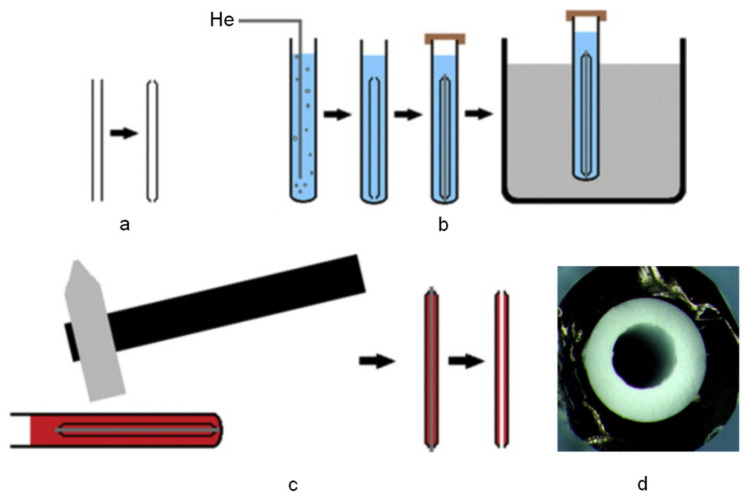
Open-tubular MIP-capillary preparation. (**a**) Both the tips of the glass-capillary were coned with flame to the diameter size of the desired metal rod. (**b**) The prepared assembly was placed in a bigger capillary that contained polymer mixture. (**c**) After the polymerization, the metal rod was removed from the polymer. (**d**) Magnified cross section of the polymer tube inside the 20 μL capillary glass. Reproduced from Figures 1 and 3 of Ref. [88] with permission from Elsevier.

Inner-surface-coated MIP capillaries have been utilized for both off-line [88,89] and on-line [90,91,92] IT-SPME. Zarejousheghani et al. [88] developed an open-tubular MIP capillary by inserting a metal rod into a glass capillary, synthesizing the MIP inside the capillary, then removing the metal rod (Figure 5a–c). The metal rod controlled the thickness of the polymer phase during in situ synthesis resulting in a robust and mechanically stable MIP tube (Figure 5d). This technique was applied to a selective off-line IT-SPME method for 4-nitrophenol in water. Souza et al. [89] synthesized a new molecule modified with RAM in an open-tubular fused silica capillary imprinted polymer (RAM-MIP). This extraction capillary, directly connected to a syringe for IT-SPME, was applied to the determination of parabens in breast milk by UHPLC–MS/MS analysis. Asiabia et al. [90] prepared nanostructured copolymers composed of polypyrrole-EGDMA copolymers on the inner surface of stainless-steel tubes through electrochemical synthesis. These were applied to selective analysis of indomethacin in urine and plasma by on-line MIP IT-SPME HPLC-UV. Recently, Song et al. [92] synthesized MIPs mixed with magnetic nanoparticles (Fe_3_O_4_) in situ in a capillary using 2,4-dinitroaniline (2,4-DNA) as a model template. A magnetic coil wrapped around this MIP-based microextraction tube generated a variable magnetic field during on-line IT-SPME, selectively adsorbing and desorbing 2,4-DNA in environmental water.

**Figure 6:**
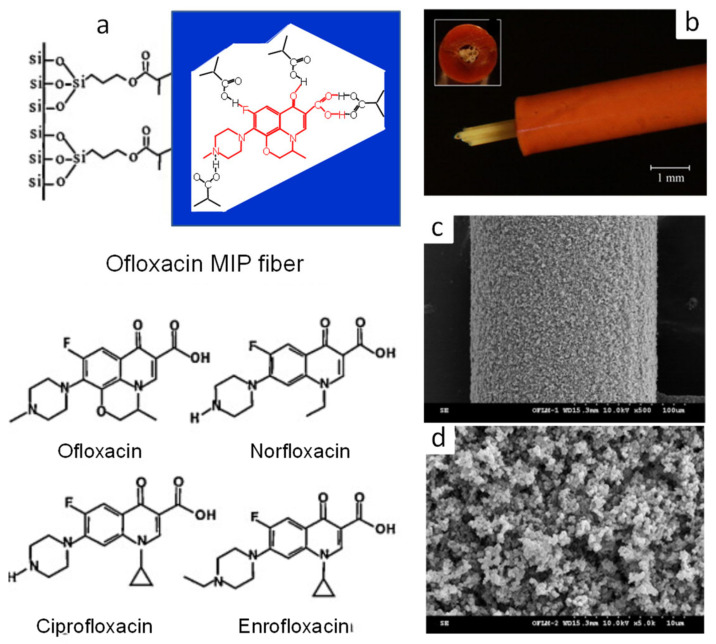
MIP fibers and fiber-packed tubes that recognize fluoroquinolones. (**a**) The chemical structure of fluoroquinolones and the schematic diagram of the resultant MIP structure. (**b**) Micrograph of the multiple-fiber-packed tube. (**c**,**d**) SEM images of the ofloxacin MIP fiber. Reproduced from Figures 1–3 of Ref. [94] with permission from Elsevier.

Packed MIP capillaries have been employed in on-line IT-SPME using both particle-packed capillaries [87,93] and fiber-packed capillaries [94]. Chaves et al. [93] synthesized protein-template MIPs using a sol-gel method and developed a gentle template removal method using proteases. MIP particles of interferon alpha 2a were packed in PEEK tubes, leading to the development of an on-line IT-SPME HPLC fluorescence detection method. These MIPs functioned like other selective interferon alfa 2a immobilized phases (e.g., immunosorbents and access-limiting substances), were robust, easy to handle, inexpensive to synthesize, and applicable to the analysis of small plasma samples (50 μL). Hu et al. [94] developed a multi-fiber-packed on-line IT-SPME method, where MIP fibers were packed longitudinally in PEEK tubes (Figure 6). This design reduced back pressure, accelerated reaction rates, and increased extraction capacity by increasing the coating volume. The method was applied to HPLC-UV analysis of fluoroquinolones and sulfonamides in animal food samples, achieving high selectivity and sensitivity.

**Figure 7:**
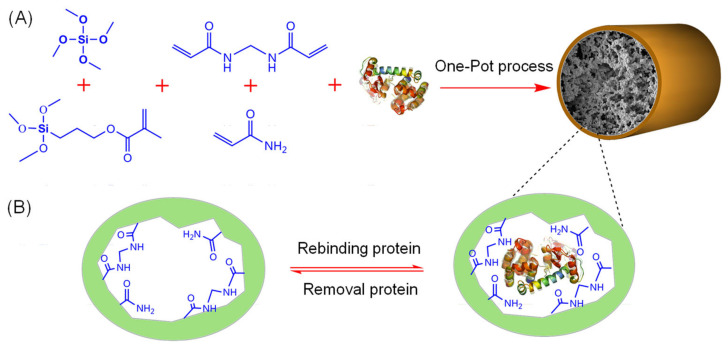
Schematic representation of (**A**) one-pot synthesis of protein-imprinted hybrid monolithic column and (**B**) the recognition mechanism between template protein and functional monomers. Reproduced from Figure S1 of Ref. [98] with permission from Elsevier.

**Figure 8:**
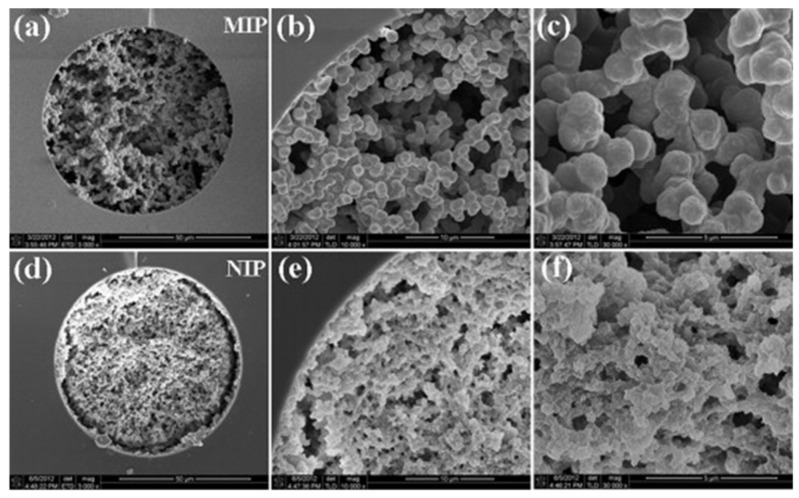
SEM images of the Lyz-MIP (**a**–**c**) and NIP (**d**–**f**) hybrid monolithic columns. (**a**,**d**) 3000×, (**b**,**e**) 10,000×, (**c**,**f**) 30,000×. Reproduced from Figure 1 of Ref. [98] with permission from Elsevier.

Monolithic MIP capillaries have been used in both off-line [95,96] and on-line [97,98,99,100,101,102] IT-SPME. Lei et al. [96] used surface imprinting technology to develop a dummy template (Pro-Tyr-Ile-Leu) to fabricate novel MIP monoliths in capillaries. This off-line method, directly connected to a syringe, exhibited high selectivity for target peptides and low detection limits, and was applied to selective MIP IT-SPME HPLC-UV analysis of neuropeptide neurotensin and neuromedin N in human plasma samples. Lin et al. [98] fabricated a molecularly imprinted inorganic-organic hybrid monolithic capillary using lysozyme as a template in combination with a rigid silica matrix and a flexible organic hydrogel by one-pot process (Figure 7). The resulting highly porous, uniform monolithic matrix is firmly fixed to the inside of the capillary (Figure 8), forming a stable, accessible recognition site, and promoting template re-binding with high imprint coefficients. This method allows selective separation of lysozyme from egg white and human serum through on-line coupling of MIP IT-SPME and pressurized capillary electrochromatography (pCEC)-UV. Szumski et al. [100] synthesized MIPs with high imprinting factors in poly(trimethylolpropane trimethacrylate) (poly-TRIM) core monoliths using 5,7-Dimethoxycoumarin as a dummy template of aflatoxin. This technique was successfully used in the selective separation of aflatoxins B1, B2, G1, and G2 in aqueous solution by microLC-laser induced fluorescence detection. Additionally, Marchioni et al. [102] synthesized durable MIP monoliths in a fused silica capillary using hydrogenated cannabidiol (CBD) as a dummy template through in situ polymerization. An automated analytical method using online coupling of IT-SPME and UHPLC-MS/MS with this MIP adsorbent as the extraction device was successfully applied to determine cannabinoid concentrations in the plasma of patients undergoing CBD treatment.

Thus, MIPs have been widely employed as a capillary stationary phase in IT-SPME, enabling selective extraction and enrichment of various compounds in complex matrices. As discussed in Section 3, while advancements in MIP design, such as single, dual, multi-template adsorbents, and hybrid materials have improved throughput and efficiency, their application to IT-SPME requires further study. Future, developments are anticipated by integrating novel functional MIP adsorbents with IT-SPME.

## 5. Conclusions and Perspective

While highly sensitive and high-performance analytical instruments are essential for accurately and precisely measuring trace constituents in complex samples, their effectiveness largely depends on efficient sample pretreatment. The MIP-based IT-SPME method introduced in this review holds great promise as an innovative sample preparation technique due to its various advantageous features:

- High selectivity and extraction efficiency: MIPs have specific binding sites that are complementary to the structure and functional groups of the target analyte, enabling highly selective adsorption. This results in the selective extraction of analytes from complex matrix samples, reducing matrix interference and improving the sensitivity and accuracy of analysis.
- High stability and reusability: MIPs offer superior chemical and physical stability compared to biorecognition materials such as antibodies and enzymes. They can withstand harsh conditions such as exposure to organic solvents and extreme pH environments. They can also be reused after washing, making them suitable for continuous analysis in IT-SPME.
- Ease of multifunctional modification: The outer surface of MIPs can be easily customized by incorporating various monomers to provide specific functional groups. This enables the enhancement of performance by creating hybrid materials, such as the integration of magnetic nanoparticles to for improved recycling or the inclusion of RAM to limit molecular permeability. Combining MIPs with other highly porous materials can also increase surface area and porosity, facilitating mass transfer of target analytes to binding sites.
- Cost reduction due to miniaturization: IT-SPME is cost-effective due to the small extraction phase of the capillary, which allows for rapid and efficient extraction and concentration. The miniaturization of MIP capillaries significantly reduces sample volume and solvent consumption. Additionally, MIPs are relatively simple and inexpensive to synthesize, facilitating mass production.
- Labor-saving through high throughput: IT-SPME can automate the extraction, desorption, and introduction of compounds into analytical instruments on-line using column-switching techniques. This automation enables high-throughput analysis of a large numbers of samples, saving labor and time.
- Environmental friendliness: The MIP-based IT-SPME method uses minimal organic solvents, reducing health hazards to analysts, minimizing waste, and promoting environmentally friendly sample preparation. However, MIP preparation still requires the use of hazardous organic solvents.

Despite these excellent features, some challenges remain for the effective use of MIP IT-SPME in sample preparation. Issues such as unpredictable component leakage, limited reusability, scale-up constraints, and irreversible adsorption of adsorbates into MIP pores must be addressed. Additionally, IT-SPME faces challenges related to capillary structure and extraction efficiency, including capillary tube clogging, detachment of coating materials, capillary breakage, and the need to improve extraction rates and times.

To further develop and expand the use of MIP IT-SPME, future efforts should focus on overcoming these challenges, improving MIP selectivity and extraction efficiency, enhancing the multifunctionality and speed of the extraction process, facilitating on-line coupling with various analytical instruments, and developing high-throughput, highly sensitive analytical systems. Finally, we hope this review will inspire new ideas and innovations among researchers working in related scientific fields and encourage the application of MIP IT-SPME technology across a broad range of disciplines.

## Figures and Tables

**Table 1:** Various imprinting techniques for the fabrication of MIPs. Produced from Ref. [6] with permission from Elsevier.

Surface imprinting	MIPs are typically fabricated in layers on hard particles, forming high affinity recognition sites on the substrate surface. The size of the imprinting cavities on the polymer surface can be effectively controlled, and uniformly distributed sites not only increase the adsorption capacity of the MIP and improve the rebinding rates of the recognition sites to the imprinted molecules, but also enhance the adsorption and separation efficiency of the imprinted material.
Nanoimprinting	Nanoimprinting technology is used to prepare nanostructured MIPs offering advantages such as high resolution, fast processing speed, high throughput, material compatibility, and low cost. These benefits improve the adsorption capacity, recombination rate, and site accessibility of the MIPs.
Living/controlled radical polymerization technology	Common methods include nitroxide-mediated free radical polymerization, atom transfer radical polymerization, and reversible addition cleavage chain transfer polymerization. Advantages include: (1) a wide range of polymerizable monomers, controllable polymer molecular weight, and narrow molecular weight distribution; (2) mild reaction conditions, low polymerization reaction temperatures, and compatibility with various solvents; (3) structural functional control, with the use of “reactive” features and functionalized end groups allowing for the preparation of polymers with complex compositions and structures; (4) a linear increase in polymer molecular weight with the conversion rate.
Multi-template imprinting	This technique uses multiple target molecules as templates to form various recognition sites in a single polymer material. Multiple template MIPs can simultaneously recognize multiple target molecules, allowing for the concurrent extraction, separation, analysis, and detection of different species, thus greatly expanding the practical applications of MIPs.
Multi-functional monomer imprinting	Multifunctional monomer imprinting techniques utilize non-covalent bonds between two or more functional monomers and a template molecule to create different forces with selective adsorption capacity. This improves the selectivity of the MIP for the template molecule, thereby enhancing its enrichment capacity.
Dummy template imprinting	Structural analogues of the target compound are used as template molecules when the target compound is either unsuitable for use as a template molecule or susceptible to degradation.

**Table 2:** Various MIP IT-SPME methods developed for sample preparation.

Analyte	Template	Polymerization Composition ^1^ and Conditions (Monomer/Crosslinker/Initiator/Porogen) ^1^	Capillary Tube Configuration	IT-SPME Operation	Enrichment, Sensitivity ^2^	IF ^3^	Matrix	Detection ^4^	Ref.
Estrogen-related compounds	β-Estradiol	VP/EGDMA/AIBN/CH_2_Cl_2_, 50 °C for 4.5 h, template/VP/EGDMA (1:6:30). In-situ synthesis of MIP in a fused silica capillary surface by insertion of fluorocarbon yarn (65 cm × 0.20 mm).	Inner-surface-coated fused silica capillary (60 cm × 0.32 mm ID).	Draw/ejection on-line	EF: 1.9–16.4	1.8–3.6	Water	HPLC-UV	-
4-Nitrophenol	4-Nitrophenol	MAA/EGDMA/AIBN/acetonitrile, 60 °C for 4 h. In-situ synthesis of MIP in a glass capillary surface by insertion of a metal rod.	Inner-surface-coated glass-capillary (100 μL).	Syringe pump off-line	LOD: 0.33 ng mL^−1^	-	Environ-mental water	HPLC-DAD	[88]
Parabens	Benzyl-paraben	MIP: VP/EGDMA/AIBN/acetonitrile in capillary at 50 °C for 4 h, template/monomer (1:4), RAM: hydrophilic monomer GDMA/PPDS at 70 °C for 20 h.	Inner-surface-coated fused silica capillary (50 mm × 0.53 mm ID).	Syringe pump off-line	LLOQ: 3–10 ng mL^−1^	-	Breast milk	UHPLC-MS/MS	[89]
Indomethacin	Indomethacin	PY/EGDMA/AIBN/MeOH:H_2_O (2:1, *v*/*v*), cyclic voltammetry in the potential range between −1.0 and ~1.0 V during 30 cycles (scan rate: 50 mV/s).	Inner-surface-coated stainless-steel tube (10 cm × 0.75 mm ID).	Electrochemi-cal control flow-through on-line	LOD: 0.6–2.0 ng mL^−1^	-	Urine, plasma, blood	HPLC-UV	[90]
Carbamazepine	Carbam-azepine	Molecularly imprinted polypyrrole coated on CuO by electrodeposition, cyclic voltammetry in the potential range (0~+3 V during 30 cycles (scan rate: 70 mV/s).	Inner-surface-coated copper tube (10 cm × 0.78 mm ID).	Flow-through on-line	LOQ: 0.1 ng mL^−1^	-	Urine, plasma	HPLC-UV	[91]
2,4-Dinitroaniline (2,4-DNA)	2,4-DNA	VI/EDMA/AIBN/1-propanol:1,4-butanediol (1:1)/Fe_3_O_4_ nanoparticles pre-modified with γ-MAPS at 70 °C for 12 h, template/VI/EDMA (4:1:4).	Inner-surface-coated fused silica capillary (2 cm × 0.53 mm ID).	Magnetic field control flow-through on-line	LOD: 60 pg mL^−1^	3.1	Environ-mental water	HPLC-DAD	[92]
Propranolol	Racemic propranolol	MAA/EGDMA/AIBN/toluene at 60 °C for 18 h, template/monomer (1:2)	Particle-packed PEEK tube (80 mm × 0.76 mm ID).	Draw/ejection on-line	LOD: 0.32 μg mL^−1^	-	Serum	HPLC-UV	[87]
Interferon alpha 2a	Interferon alpha 2a	Two-step sol–gel procedure: APS/TEOS/deionized water:0.1 M HCl: absolute (EtOH) (1:1.4:1.7) + silanes, kept at room temperature for 24 h and dried at 50 °C for 48 h.	Particle-packed PEEK tube (50 mm × 0.02 inch ID).	Draw/ejection on-line	-	-	Plasma	HPLC-FD	[93]
Four fluoro-quinolone antibiotics	Ofloxacin, sulfadiazine	MAA/TRIM/AIBN/CH_3_CN:H_2_O (6:1, *v*/*v*), silica fiber (10 cm × 0.125 mm) coating in glass capillary (10 cm × 1.0 mm ID) at 60 °C for 3 h.	Fiber-packed (6 cm × 6 fibers) PEEK tube (0.5 mm ID).	Flow-through on-line	EF: 69–136, LOD: 16–110 pg mL^−1^	-	Pork liver	HPLC-UV	[94]
8-Hydroxy-2′-deoxyguanosine (8-OHdG)	Guanosine	Monolith: TEPM/methanol, 40 °C for 12 h; MIP monolith: VP/MBA/AIBN/dodecanol, in capillary at 60 °C for 18 h.	Rod monolith in fused silica capillary (50 mm × 0.53 mm ID).	Syringe pump off-line	EF: 76, LOD: 957 pg mL^−1^	-	Urine	HPLC-UV	[95]
Neurotensin, neuromedin N	Pro-Tyr-Ile-Leu	MIP monolith: MAA/EGDMA/AIBN/MeOH/acetonitrile/isooctane, 60 °C for 16 h template/MAA (1:3).	Inner-surface-coated fused silica capillary (2 cm × 0.53 mm ID).	Syringe pump off-line	LOD: 0.9–1.0 ng mL^−1^	5.7–13.4	Plasma	HPLC-UV	[96]
Anaesthetics (bupivacaine, mepivacaine, S-ropivacaine)	Bupivacaine, mepivacaine, ropivacaine	Monolith: TRIM/EDMA/BME/2,2,4-trimethylpentane/toluene (80:20, *w*/*w*), UV; MIP monolith: MAA/EDMA/AIBN/toluene, Template/MAA/EDMA (0.33:4:20), UV, 1 h	Rod monolith in UV transparent capillaries (70 mm × 0.1 mm ID).	Flow-through on-line	-	12–72	Water	HPLC-UV	[97]
Lysozyme	Lysozyme	Co-precursors: PEG/TMOS/γ-MAPS MIP hybrid monolith: co-precursors + AAm/MBA/AIBN/MeOH:H_2_O (5:3, *v*/*v*), in capillary at 40 and 60 °C for 12 h.	Rod monolith in fused silica capillary (25 cm × 75 μm ID).	Flow-through on-line	-	1.91	Serum, egg white	pCEC-UV (capLC)	[98]
Glycoprotein	Horseradish peroxidase (HRP)	Monolith: VPBA/PETA/AIBN/ethylene glycol: cyclohexanol, in capillary at 75 °C for 12 h; MIP monolith: immobilization of HRP on VPBA-based monolith and poly-dopamine (pDA) coating with DA and APS.	Rod monolith in fused silica capillary (25 cm × 75 μm ID).	Flow-through on-line	-	2.76	Serum	pCEC-UV	[99]
Aflatoxins	5,7-Dimethoxy-coumarin	Monolith: γ-MAPS/TRIM/BME or AIBN/2,2,4-trimethylpentane/toluene (80:20, *w*/*w*), UV for 1 h and 60 °C for 24 h MIP monolith: MAA/EGDMA/AIBN/toluene, template/MAA/EGDMA (0.3:4:20), UV, 1 h	Rod monolith in UV transparent fused capillary (70 mm × 0.1 mm ID).	Flow-through on-line	-	-	Water	MicroLC-LIF	[100]
Cocaine and its metabolite	Cocaine	MIP monolith: MAA/EGDMA/AIBN/acetonitrile-isooctane (9:1), 60 °C for 24 h template/MAA/EGDMA (1:4:20).	Inner-surface-coated fused capillary (50 mm × 0.1 mm ID).	Flow-through on-line	LOD: 14.5–6.1 ng mL^−1^	2.2–3.2	Plasma, saliva	NanoLC-UV	[101]
Cannabinoids	Hydrogenated cannabidiol	MIP monolith: MAA/EGDMA/AIBN/CH_2_Cl_2_, 60 °C for 24 h, template/MAA (1:3).	Inner-surface-coated fused capillary (10 cm × 0.53 mm ID).	Flow-through on-line	LLOQ: 10 ng mL^−1^	-	Plasma	UHPLC-MS/MS	[102]

^1^ Functional monomer: MAA, methacrylic acid; VP, 4-vinylpyridine; AAm, acrylamide: APS, 3-aminopropyltriethoxysilane; VPBA: 4-vinylphenylboronic acid; PY, pyrrole; VI, vinylimidazole. Crosslinker: EGDMA, ethylene glycol dimethacrylate; EDMA, ethylene dimethacrylate; MBA, N,N-methylene bisacrylamide; TRIM, trimethylolpropane trimethacrylate; TEOS, tetraethoxysilane; PETA: pentaerythritol triacrylate; TEPM, 3-(triethoxysilyl)propyl methacrylate; GDMA, glycerol dimethacrylate; GMA, glycidyl methacrylate. Initiator: AIBN, 2,2′-azobis-isobutyronitrile; BME, benzoin methyl ether; APS, ammonium persulfate; PPDS, potassium peroxodisulfate; A4-CA, 4,4′-azobis (4-cyanovaleric acid). Co-precursors PEG, poly(ethylene glycol; TMOS, tetramethyloxysilane; γ-MAPS, 3-methacryloxypropyltrimethoxysilane; ^2^ EF: enrichment factor, LOD: limit of detection; LLOQ: lower limit of quantification; LOQ: limit of quantification; ^3^ IF: imprinting factor (MIP/NIP, molecularly imprinted capillary/non-imprinted capillary). ^4^ pCEC: pressurized capillary electrochromatography; DAD, diode-array UV detector; LIF, laser induced fluorescence detector.

**Table 3:** Selectivity and enrichment effects of β-estradiol MIP on various compounds.

Compound	IF ^1^	EF ^2^	Compound	IF	EF	Compound	IF	EF
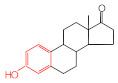 Estrone	2.78	16.4	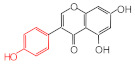 Genistein	3.56	12.2	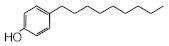 Nonylphenol	1.39	4.27
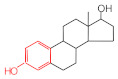 β-Estradiol	2.35	3.60	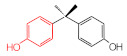 Bisphenol A	2.64	5.26	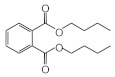 Di-n-butyl phthalate	1.21	2.54
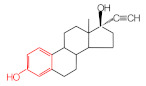 Estriol	2.43	1.93	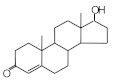 Progesterone	1.50	2.63	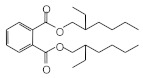 Di-2-ethylhexyl phthalate	0.86	1.45
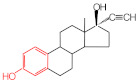 Ethinylestradiol	2.29	5.60	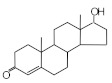 Testosterone	0.96	1.03	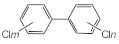 Polychlorinated biphenyl (PCBs)	0.92	13.7
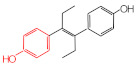 Diethylstilbestrol (DES)	1.79	2.63	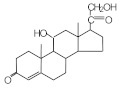 Corticosterone	0.77	0.77	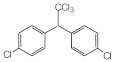 Dichlorodiphenyl- trichloroethane (DDT)	1.16	19.0

^1^ IF: Imprinting factor (peak area ratio obtained using MIP and NMIP). ^2^ EF: Enrichment factor (peak area ratio obtained using MIP IT-SPME and direct injection).

## Data Availability

No new data were created or analyzed in this study.
